# Supplementary material for: Ensuring That Fundamentals of Quantitative Microbiology Are Reflected in Microbial Diversity Analyses Based on Next-Generation Sequencing
Source: Front Microbiol. 2022 Mar 1;13:728146. doi: 10.3389/fmicb.2022.728146 (PMC8921663; doi:10.3389/fmicb.2022.728146)
Supplement: Supplementary file 1 [file Data_Sheet_1.docx]

Supplementary Material

# Supplementary Data

Table S1: Simulated data based on drawing tiles from the game Scrabble^TM^ with replacement

| Tile Variant | True Proportion | Library Size | | | | | |
| --- | --- | --- | --- | --- | --- | --- | --- |
|  |  | 25 | 50 | 100 | 200 | 500 | 1000 |
| A | 0.09 | 1 | 6 | 8 | 18 | 52 | 101 |
| B | 0.02 | 0 | 1 | 2 | 4 | 7 | 12 |
| C | 0.02 | 1 | 1 | 1 | 6 | 7 | 16 |
| D | 0.04 | 1 | 0 | 5 | 6 | 20 | 40 |
| E | 0.12 | 4 | 5 | 7 | 29 | 70 | 118 |
| F | 0.02 | 0 | 0 | 2 | 6 | 13 | 22 |
| G | 0.03 | 0 | 2 | 5 | 8 | 10 | 28 |
| H | 0.02 | 1 | 2 | 1 | 3 | 5 | 20 |
| I | 0.09 | 2 | 9 | 8 | 13 | 44 | 97 |
| J | 0.01 | 0 | 0 | 2 | 2 | 1 | 11 |
| K | 0.01 | 0 | 0 | 2 | 4 | 3 | 10 |
| L | 0.04 | 1 | 2 | 1 | 3 | 28 | 52 |
| M | 0.02 | 0 | 1 | 1 | 3 | 8 | 14 |
| N | 0.06 | 0 | 5 | 7 | 10 | 27 | 51 |
| O | 0.08 | 2 | 3 | 13 | 17 | 43 | 77 |
| P | 0.02 | 1 | 1 | 2 | 6 | 9 | 18 |
| Q | 0.01 | 0 | 0 | 3 | 1 | 4 | 8 |
| R | 0.06 | 2 | 4 | 4 | 13 | 30 | 65 |
| S | 0.04 | 2 | 2 | 5 | 6 | 21 | 44 |
| T | 0.06 | 2 | 3 | 5 | 14 | 23 | 50 |
| U | 0.04 | 1 | 0 | 6 | 6 | 31 | 40 |
| V | 0.02 | 1 | 1 | 1 | 4 | 10 | 28 |
| W | 0.02 | 2 | 1 | 2 | 4 | 6 | 17 |
| X | 0.01 | 1 | 1 | 3 | 3 | 3 | 11 |
| Y | 0.01 | 0 | 0 | 1 | 4 | 8 | 19 |
| Z | 0.01 | 0 | 0 | 1 | 2 | 4 | 9 |
| Blank | 0.02 | 0 | 0 | 2 | 5 | 13 | 22 |

# R code: Simulation analysis to determine if clustering or amplification affect multinomial model for amplicon sequencing

Note: If $X\sim Poisson\left( \mu\right)$ and $\mu\sim gamma\left( \rho,\lambda\right)$ with shape parameter $\rho$ and scale parameter $\lambda$, then $X$ is negative binomially distributed with $p\left( x \right)=\frac{\Gamma\left( x+\rho\right)}{\Gamma\left( \rho\right)x!}\left( \frac{1}{1+\lambda} \right)^{\rho}\left( \frac{\lambda}{1+\lambda} \right)^{x}$. This distribution has mean $E\left[ X \right]=\rho\lambda$ and variance $Var\left[ X \right]=\rho\lambda\left( 1+\lambda\right)$ so that $\lambda$ relates to the overdispersion of the distribution relative to a Poisson distribution. The R function rnbinom has parameters size = $\rho$ and prob = $\left( \frac{1}{1+\lambda} \right)$.

### Install package ggplot2 and add to library

install.packages("ggplot2")

library(ggplot2)

### Fix randomization seed for reproducibility

set.seed(20210528)

### ANALYSIS #1 – CLUSTERING

### Generate dataset (10^6 draws) from A0 ~ binomial(n=6, p=2/3) & B0 = 6 – A0

### Special case of multinomial relative abundance model in which A:B = 2:1

A0 <- rbinom(1000000, 6, 2/3)

B0 <- 6 - A0

### Construct a negative binomial model as a function of the mean (mu=rho*lambda)

### and overdispersion relative to Poisson (lambda) in which A:B = 2:1

### Simple model with microorganisms clustering within but not between varieties

### Generate dataset (10^6 draws) from A1 ~ negbin(size=40,000, prob=1/10,001)

### & B1 ~ negbin(size=20,000, prob=1/10,001)

### With lambda = 0.0001, variants are essentially not clustered and the negative

### binomial model is approximately Poisson(mu)

muA <- 4

lambdaA <- 0.0001

rhoA <- muA / lambdaA

sizeA <- rhoA

probA <- 1 / (1 + lambdaA)

muB <- 2

lambdaB <- 0.0001

rhoB <- muB / lambdaB

sizeB <- rhoB

probB <- 1 / (1 + lambdaB)

A1 <- rnbinom(1000000, size = sizeA, prob = probA)

B1 <- rnbinom(1000000, size = sizeB, prob = probB)

SUM1 <- A1 + B1

### Create data frame of subset of simulations in which SUM1 = 6

Poisson <- subset(data.frame(A1, B1, SUM1), SUM1 == 6)

### Generate dataset (10^6 draws) from A2 ~ negbin(size=4/3, prob=0.25)

### & B2 ~ negbin(size=2/3, prob=0.25)

### With lambda = 3, variants are substantially clustered

muA <- 4

lambdaA <- 3

rhoA <- muA / lambdaA

sizeA <- rhoA

probA <- 1 / (1 + lambdaA)

muB <- 2

lambdaB <- 3

rhoB <- muB / lambdaB

sizeB <- rhoB

probB <- 1 / (1 + lambdaB)

A2 <- rnbinom(1000000, size = sizeA, prob = probB)

B2 <- rnbinom(1000000, size = sizeB, prob = probB)

SUM2 <- A2 + B2

### Create data frame of subset of simulations in which SUM2 = 6

Cluster <- subset(data.frame(A2, B2, SUM2), SUM2 == 6)

### Construct graph of relative frequency of A0, A1, and A2

A0relfreq <- table(A0)/1000000

A1relfreq <- table(Poisson$A1)/length(Poisson$A1)

A2relfreq <- table(Cluster$A2)/length(Cluster$A2)

Legend <- rep(c("A0", "A1", "A2"), each = 7)

X <- rep(c(0, 1, 2, 3, 4, 5, 6), times = 3)

Y <- c(A0relfreq, A1relfreq, A2relfreq)

summary1 <- data.frame(Y, Legend, X)

ggplot(summary1, aes(fill = Legend, y = Y, x = X)) +

labs(y = "Relative Frequency", x = "Count A") +

scale_fill_manual(values = c("#66E199", "#66CCFF", "#FF33CC")) +

geom_bar(position = "dodge", stat = "identity")


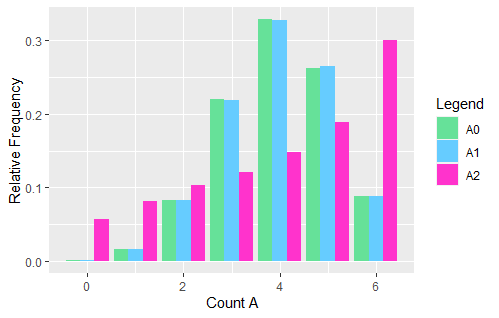


### Relative frequencies of A0 and A1 are similar, suggesting that random sampling ### error following a Poisson model is compatible with the multinomial relative

### abundance model. Relative frequencies of A0 and A2 are strongly dissimilar,
### proving that random sampling error following a negative binomial model is
### incompatible with the multinomial relative abundance model.

### ANALYSIS #2 - AMPLIFICATION

### Generate dataset (10^6 draws) from A3 ~ binomial(n=4, p=2/3) & B3 = 4 – A3

### Special case of multinomial relative abundance model in which A:B = 2:1

A3 <- rbinom(1000000, 4, 2/3)

B3 <- 4 - A3

### Amplify A3 and B3 with a 50% success rate

A3Amp <- A3 + rbinom(1000000, A3, 0.5)

B3Amp <- B3 + rbinom(1000000, B3, 0.5)

SUM3 <- A3Amp + B3Amp

### Create data frame of subset of simulations in which SUM3 = 6

Amplify <- subset(data.frame(A3Amp, B3Amp, SUM3), SUM3 == 6)

### Construct graph of relative frequency of A0 and A3

A3relfreq <- table(Amplify$A3Amp)/length(Amplify$A3Amp)

Legend <- rep(c("A0", "A3"), each = 7)

X <- rep(c(0, 1, 2, 3, 4, 5, 6), times = 2)

Y <- c(A0relfreq, A3relfreq)

summary2 <- data.frame(Y, Legend, X)

ggplot(summary2, aes(fill = Legend, y = Y, x = X)) +

labs(y = "Relative Frequency", x = "Count A") +

scale_fill_manual(values = c("#66E199", "#66CCFF")) +

geom_bar(position = "dodge", stat = "identity")


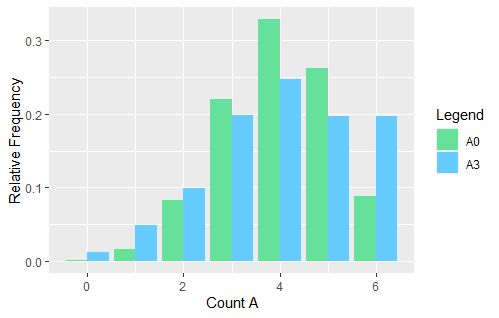


### Relative frequencies of A0 and A3 are strongly dissimilar, proving that
### amplification is incompatible with the multinomial relative abundance model.

# OpenBUGS code: Bayesian analysis of Shannon index with multinomial relative abundance data

p[i]: fraction of sequences of type i - STOCHASTIC (parameter of interest)

m: number of unique sequence types – SPECIFIED

alpha[i]: parameter of Dirichlet prior for sequences of type I - SPECIFIED

X[i]: count of sequences of type i - STOCHASTIC (measured data)

N: library size (sum of observed sequence counts) - DETERMINISTIC

PlnP[i]: p*ln(p) of type i - DETERMINISTIC

Shannon: Shannon index evaluated using posterior proportions - DETERMINISTIC

model {

# Define prior that is equal for all fractions in the multinomial model

p[1:m] ~ ddirich(alpha[])

# Define likelihood

X[1:m] ~ dmulti(p[1:m], N)

N <- sum(X[])

for (i in 1:m) {

PlnP[i] <- p[i]*log(p[i])

}

Shannon <- -sum(PlnP[])

}

DATA - Simple example with four variants

list(m=4)

alpha[] X[]

1 40

1 30

1 20

1 10

END

# R code: Generation of Figure 4 (sample-level Shannon index simulation analysis)

### Generate population (based on Scrabble)

Variants <- c("A", "B", "C", "D", "E", "F", "G", "H", "I", "J", "K", "L", "M",

"N", "O", "P", "Q", "R", "S", "T", "U", "V", "W", "X", "Y", "Z", "BLANK")

Fractions <- c(0.09, 0.02, 0.02, 0.04, 0.12, 0.02, 0.03, 0.02, 0.09, 0.01, 0.01,

0.04, 0.02, 0.06, 0.08, 0.02, 0.01, 0.06, 0.04, 0.06, 0.04, 0.02, 0.02, 0.01,

0.02, 0.01, 0.02)

ShannonPop <- -sum(Fractions * log(Fractions))

### Generate 1000 samples with a library size of 25, compute Shannon Index

set.seed(44335) # Fixed seed for randomizations

Data25 <- rmultinom(n = 1000, size = 25, prob = Fractions)

Shannon25 <- -colSums(Data25 / 25 * log(Data25 / 25), na.rm = TRUE)

### Generate 5 samples with larger library sizes, compute Shannon index

Data50 <- rmultinom(n = 1, size = 50, prob = Fractions)

Data100 <- rmultinom(n = 1, size = 100, prob = Fractions)

Data200 <- rmultinom(n = 1, size = 200, prob = Fractions)

Data500 <- rmultinom(n = 1, size = 500, prob = Fractions)

Data1000 <- rmultinom(n = 1, size = 1000, prob = Fractions)

ShannonRaw50 <- -sum(Data50 / 50 * log(Data50 / 50), na.rm = TRUE)

ShannonRaw100 <- -sum(Data100 / 100 * log(Data100 / 100), na.rm = TRUE)

ShannonRaw200 <- -sum(Data200 / 200 * log(Data200 / 200), na.rm = TRUE)

ShannonRaw500 <- -sum(Data500 / 500 * log(Data500 / 500), na.rm = TRUE)

ShannonRaw1000 <- -sum(Data1000 / 1000 * log(Data1000 / 1000), na.rm = TRUE)

### Repeatedly rarefy data with large library sizes to rarefied library size 25

library(extraDistr)

Rarefy50 <- rmvhyper(nn = 1000, n = t(Data50), k = 25)

Shannon50 <- -rowSums(Rarefy50 / 25 * log(Rarefy50 / 25), na.rm = TRUE)

Rarefy100 <- rmvhyper(nn = 1000, n = t(Data100), k = 25)

Shannon100 <- -rowSums(Rarefy100 / 25 * log(Rarefy100 / 25), na.rm = TRUE)

Rarefy200 <- rmvhyper(nn = 1000, n = t(Data200), k = 25)

Shannon200 <- -rowSums(Rarefy200 / 25 * log(Rarefy200 / 25), na.rm = TRUE)

Rarefy500 <- rmvhyper(nn = 1000, n = t(Data500), k = 25)

Shannon500 <- -rowSums(Rarefy500 / 25 * log(Rarefy500 / 25), na.rm = TRUE)

Rarefy1000 <- rmvhyper(nn = 1000, n = t(Data1000), k = 25)

Shannon1000 <- -rowSums(Rarefy1000 / 25 * log(Rarefy1000 / 25), na.rm = TRUE)

### Plotting

boxplot(Shannon25, Shannon50, Shannon100, Shannon200, Shannon500, Shannon1000,

xlab = "Library Size", ylab = "Shannon Index", ylim = c(1.5,3.5), las = 1,

names = c("25*", "50", "100", "200", "500", "1000"))

### Add sample Shannon index values

abline(ShannonPop, 0, col = "red", lty = 2)

points(2, ShannonRaw50, col = "red", pch = 16, cex = 1.25)

points(3, ShannonRaw100, col = "red", pch = 16, cex = 1.25)

points(4, ShannonRaw200, col = "red", pch = 16, cex = 1.25)

points(5, ShannonRaw500, col = "red", pch = 16, cex = 1.25)

points(6, ShannonRaw1000, col = "red", pch = 16, cex = 1.25)
